# Diphtheria Antitoxin, with Report of Cases

**Published:** 1897-11

**Authors:** Henry R. Slack

**Affiliations:** LaGrange, Ga.


ATLANTA

Medical and Surgical Journal.

VOL. XIV. NOVEMBER, 1897. No. 9.

L. B. GRANDY, M.D., MANAGING EDITOR.

M. B. HUTCHINS, M.D., BUSINESS MANAGER.

COLLABORATORS:
A. W. CALHOUN, M.D., LL.D., VIRGIL O. HARDON, M.D., FLOYD W. McRAE, M.D., A. W. STIRLING, M.D., JOSEPH EVE ALLEN, M.D., AUGUSTA, GA., AND HENRY R. SLACK, PH.M., M.D., LAGRANGE, GA.

### ORIGINAL COMMUNICATIONS.

### DIPHTHERIA ANTITOXIN, WITH REPORT OF CASES.

BY HENRY R. SLACK, PH.M., M.D.,
LAGRANGE, GA.

There is no disease of childhood so dreaded by parent and physician as diphtheria. This fear is well founded, for until 1895 the mortality from this fatal malady, in the best hospitals, ranged from forty to sixty per cent. In country practice it is sometimes even worse—a death-rate nearly four times as great as yellow fever during the last epidemics, yet the latter disease paralyzes commerce and calls forth the strong arms of both national and state government to stamp it out; but aside from occasionally breaking up a school, diphtheria receives very little attention from the public press.

Diphtheria is to be feared too, because, unlike yellow fever, scarlet fever and other contagious diseases, it has, according to

Osler and other high authorities, steadily increased during the last decade. Other contagious diseases have apparently diminished in the last ten years, but this emissary of death has been more active. These facts have made the advanced medical workers hail with delight any rational treatment that promised an improvement on the old methods. These were so inefficient that in the Hôpital Trousseau, out of 520 cases, 316 died—a mortality of 60 per cent.

### DISCOVERERS.

The man who discovers a cure for diphtheria deserves a place beside Jenner, who has robbed small-pox of its terrors, and Pasteur, whose discovery has saved many from the horrid death by hydrophobia. For this honor the closing years of the nineteenth century present two candidates—Roux of France, and Behring of Germany. These two investigators, the former a pupil of the great Pasteur, working independently of each other, discovered the antitoxic effects of blood-serum from immunized animals upon other animals suffering with diphtheria. The animals experimented upon were chiefly guinea-pigs. This serum they called Diphtheria Antitoxin.

### PREPARATION.

It is prepared in Roux's laboratory in Paris as follows: A large flat bottom flask, with lateral tubulature, is filled with an alkaline pepton-bouillon, and after sterilization the bouillon is inoculated with culture of diphtheria bacillus. The flask is placed in a thermostat for twenty-four hours for the purpose of starting the growth. It is then connected with an aspirator, by means of which moist air is made to pass through the flask. After the flask has been kept at a temperature of 37° C. (98.6° F.) for three or four weeks, the cultures are filtered into sterilized flasks and standardized. Immunity in the animal, the horse being used exclusively, is brought about by injecting the toxin, in gradually increasing doses, until 250 c.c. (8.3 oz.) are introduced, at which point the horse will be immune against diphtheria. The serum of such a horse has antitoxic properties, 1 grain being usually sufficient to protect 50,000 grams of guinea-pig against a fatal quantity of culture of diphtheria-bacilli. The blood is taken from the jugular vein of the horse, under antiseptic precautions, to the amount of 6 or 8 liters ($1\frac{1}{2}$ or 2 gals.) at a time. It is then set aside in a cool place and the serum collected after twenty-four hours. This serum is standardized according to Behring, so that one-tenth of one cubic centimeter will counteract ten times the minimum dose of diphtheria poison fatal to a guinea-pig weighing 300 grams. One cubic centimeter of this normal serum he calls an antitoxin unit. The serum is prepared by Parke, Davis & Co. in practically the same way, and comes on the market in five strengths—No. 0, 250 Antitoxic units; No. 1, 500; No. 2, 1000; No. 3, 1500, and No. 4, 2000. The horse is preferred by Roux for two reasons: first, horse serum, even in large doses, does no harm to man; second, because of the large amount of antitoxic serum he furnishes, and of the rapidity with which it can be obtained. Roux's paper before the International Congress of Hygiene, 1894, at Buda-Pesth, is comprehensive and covers the whole subject from the cultivation of the bacilli to the results of serum therapy.

### RESULTS.

Statistics are usually dry and often misleading, but in the case of diphtheria antitoxin they are bright and interesting, and cheer the physician as he hurries to the bedside of the little sufferer. He can now make a much more hopeful prognosis of the case, for he knows how greatly the mortality has been reduced by promptly using antitoxin.

In the Hôpital Trousseau, where the death-rate was 60 per cent. under the old methods, after introducing antitoxin it fell to only 14.7 per cent. In the Hôpital des Infants-Malades, in Paris, the mortality was reduced from 51.7 per cent. to 24.3, with no bad results that could be ascribed to antitoxin except erythema. It may be objected that these are all reports from foreign hospitals, so we will come nearer home. In the winter of 1895-96 I first saw antitoxin used, and heard Dr. Booker, of the Johns Hopkins Hospital, tell his class that this was his ninth case without a death or any bad effects except a slight rash in one instance. This was a delightful change, due to the progress of medicine, for only five years before I heard an eminent medical teacher say he had lost 7 out of 10 cases treated. Returning home a very successful physician told me he had never seen but one case recover in all his practice.

### IN PRIVATE PRACTICE.

The committee from the American Pediatric Society, appointed to collect statistics of the use of antitoxin in private practice, received reports from 615 physicians in all parts of the United States and Canada. The committee in its report gave the benefit of the doubt against antitoxin, as they excluded all doubtful cases that recovered when microscopical examination was not made, and included all doubtful cases that died. Even with these odds against it, in 5,974 cases reported the general mortality was only 13.3 per cent. If those in a moribund condition, of dying within 24 hours of the injection, be excluded, it was but 8.8 per cent.; 4,120 cases injected before the fourth day gave death-rate of 7.3 per cent. The mortality, in 1,443 cases injected on or after the fourth day, was 27 per cent. No new cases of sudden death after the injection were returned, and but 19 cases seemed to be uninfluenced favorably by the injection, and 9 of these were of doubtful diagnosis; 7 were complicated with other diseases, and in 2 the serum was of doubtful strength and value. Only 3 cases were reported by the observer as being made worse by the treatment. In a large number the heart's action was improved after the injection.

### IMMUNIZATION.

Some physicians, due to prejudice or ignorance of serum therapy, say that the development of diphtheria can't be prevented. Right here the results given by the use of antitoxin are, if possible, more satisfactory than in the treatment. Behring reports 10,000 cases immunized, only 10 of which contracted diphtheria. The New York Infant Asylum, Child's Hospital, Bellevue and others, report 1,043 cases immunized with doses ranging from 50 to 600 antitoxin units. Of this entire number only 7 cases developed diphtheria within 30 days, and 13 after 30 days. Whereas, before the use of antitoxin as a preventive, 134 cases developed in about the same time.

With these facts in view, I fully determined to use antitoxin in treating my first case of diphtheria, and provided myself with an antitoxin syringe before leaving the Johns Hopkins Hospital. Coming to Atlanta I saw Dr. Floyd W. McRae, Secretary of the Board of Health, in reference to obtaining fresh antitoxin through the board of health.

CASES.

*Case 1.*—July 15, 1897. A. L., age four years, boy; rather delicate child. Family history negative, except several have had erysipelas. Has had measles, whooping-cough and tonsillitis. Was taken on 3d with fever and sore throat. When I saw him temperature was 102°, tonsils swollen and covered with grayish white patches of membrane, as were also posterior portion of uvula and part of pharynx. Throat almost closed. Tongue coated and breath fetid. Smeared number of cover slips with mucus from membrane, wiped off with sterilized cotton swob. Then treated throat locally with hydrogen peroxide solution, which removed membrane, but left a raw, bleeding surface. Gave solution salicylate ammonia every three hours.

July 6th. Microscopical examination showed Klebs-Loeffler bacilli in great numbers. Dr. E. A. Cason called in consultation. He concurred in use of antitoxin. Temperature 101, pulse 120, weak. Injected 750 units antitoxin and removed exudate with hydrogen peroxide. Had child frequently gargle with solution of borolyptol.

July 7th. Patient rested well all night. Temperature 99.5; pulse stronger, membrane peeling off, swelling subsiding.

July 8th. Temperature 98.2, tonsils not half so large, with very little exudate; feels much better and wants to get up. Continued mouth-wash of borolyptol and gave whisky in milk.

July 9th. Temperature normal; no membrane; up playing about.

*Case 5.*—September 9, 1897. M. F., age six; girl. Had brother to die of diphtheria three weeks before. Family history negative; well nourished child. Temperature 100; had been 102.6 before calomel acted, given by her father, who is a physician ; pulse 120. Examination showed inflamed and swollen tonsils, but no exudate that might not have come from tonsillar crypts.

Sept. 10th. Called to see patient again. Now much worse and parents greatly alarmed, as same symptoms appearing as in case of son who died. Temperature 103; pulse 120, weak; grayish white exudate on left tonsil. Took culture and smeared several slides, but did not wait for results of bacteriological examination before injecting 750 units of antitoxin. In one hour her temperature had fallen to 102.2 ; pulse to 112 and good volume, and patient was sleeping quietly. Hydrogen peroxide and borolyptol used locally.

Sept. 11th. Rested fairly well. Temperature 99.4; pulse 120 but full; tonsils diminished in size and very little exudate. Continued local treatment. Complains of tenderness where needle was inserted.

Sept. 12th. Doing nicely. Temperature 99; pulse 112; inflammation of tonsils gone; appetite returned. Dismissed case.

*Cases 2 and 3.*—Both in same family; were seen in consultation with Dr. E. A. Cason. Antitoxin was used and both recovered. A brother of theirs, whom I did not see, but who was inoculated on fifth day, died.

*Case 4.*—Negro child, four years, a patient of Dr. Cason, was seen on second day and presented a typical, clinical picture of diphtheria. One thousand units were injected, and he made a rapid and uneventful recovery, except slight paralysis of larynx that interferes somewhat with his articulation. No microscopical examination was made in this case, but was in each of the other four, and Klebs-Loeffler bacillus found. There were five other children in the family where first case occurred. I injected each with antitoxin, doses ranging from 50 to 250 units, according to age, to immunize them. They suffered absolutely no inconvenience from the treatment, not even a rash appearing, and none had diphtheria.

In case 5 I used 50 units to immunize a little sister of five months. This caused an elevation of 1 degree and some soreness that made her restless and fretful for about twenty-four hours, but no untoward symptoms.

In treating these cases we used diphtheria antitoxin of both foreign and domestic makes, but I prefer that prepared by Parke, Davis & Co. for two reasons: First, it is put up in hermetically sealed glass bulbs; second, because it can be obtained fresher, which increases its reliability. Roux says: "The serum may cause transient eruptions, but it is not dangerous; it does not kill." He bases this statement on the fact that the serum has been used in over 100,000 cases and it has charged to it only two deaths (Prof. Langersham's child and Miss Valentine). It would be almost impossible to inject hypodermically 10 c.c. of pure water into each of 100,000 persons without a larger fatality.

### CONCLUSION.

From my experience in treating diphtheria with antitoxin, and the statistics gathered by such men as Welch, Biggs and others, I unhesitatingly recommend its use, not only as treatment, but as a preventive.

Antitoxin should be administered as early as possible on a clinical diagnosis, not waiting for the bacteriological examination, as twenty-four hours lost may mean a life sacrificed. The dose, according to report of the committee of the American Pediatric Society, should be 1,000 to 2,000 units, according to the severity of the case, to be repeated in eighteen to twenty-four hours if not improved, and a third dose may be given.

Use the most concentrated strength of an absolutely reliable preparation, and the result will be satisfactory to your patient, the parents and yourself.

### LITERATURE.

Osler, Practice Medicine, 1895; Bulletin Johns Hopkins Hospital, July–August, 1895; Journal American Medical Association, Vols. 26, 27 and 28; American Journal Medical Science, October, 1894; British Medical Journal, August, 1895; Berliner Klinsche Wochenschrift, September, 1895; Lyon Médicale, 1895; Medical News, November, 1895; Medical Record, June, 1896; Munich Medical Wochens., November, 1894; University Medical Magazine, March, 1895; Year Book of Medicine, Gould, 1896 and 1897.

